# Nervous Diseases Caused by Alcoholic and Metalic Poisoning

**Published:** 1908-07

**Authors:** J. Cheston King

**Affiliations:** Atlanta, Ga.


### NERVOUS DISEASES CAUSED BY ALCOHOLIC AND METALIC POISONING.*

BY J. CHESTON KING, A. B., M. D., ATLANTA, GA.

Mr. President and Gentlemen:—The subject which I have selected for your consideration, is one which has enlisted the most careful research on the part of the medical profession, and though I may present that which is only *common-place,* yet we can least afford to disregard that which is common place, for old knowledge to new minds involves a constant renewal of youth.

I shall only discuss in a condensed manner the effect of *alcohol, lead,* and *arsenic* upon the nervous system. First, what do you understand by the nervous system? It includes the neuroglia, which supports and separates the nerve elements, the blood vessels, which penetrates and permeates the centres and the

*Read before the Fulton County Medical Association.

membranes which enclose and protects them. The effects of alcohol on the peripheral nerves are made manifest by a feeling of numbness and pins and needles in the hands and feet, followed by sharp pains, and accompanied by extreme tenderness on the nerve trunks, muscles and skin; there is often in a day or two some inco-ordination in the limbs and then a failure of muscular power. The typical places for the loss of power, are the muscles on the outer side of the leg and on the back of the forearm, supplied by the external popliteal and posterior interosseus nerves. As a result the patient has dropped-wrists and dropped feet, and finds he is unable to extend the toes, to dorso-flex the ankles, to invert or evert them; also in the upper limb, he cannot entend the fingers, the thumb, the wrist. It is almost invariably the case that the corresponding parts of the right and left sides are effected at the same time, and the symetrical flexion of the fingers and wrists and the extension of the ankles with the foot in a line with the tibia are very characteristic. On the sensory side, besides the tingling and extreme tenderness, there is some anaesthesia of the hands and feet, which spreads up the outer side of the leg and the radial borders of the forearm. Inco-ordination of the arms and legs is sometimes met with. As the disease progresses, the muscles below the knee and elbow are affected, the extensors and then the flexors of the knee and elbow may be paralysed, and subsequently the muscles of the hips and shoulders. In very severe cases, the trunk muscles, the intercostals, the diaphragm, and the heart. The muscles waste very much, and in three weeks time electrical changes occur, so that they seem to contract to the faradic current and give the reaction of degeneration with the constant current, while the nerves lose all response to electricity. If only one set of muscles, viz: The extensors, be paralysed, the unapposed flexors produce a contracture with deformity of the joints.

The knee jerks are lost if the disease involves the extensors of the knee.

The plantor skin reflex is as a rule lost. Sensory disturbances increase along with motor, which they often exceed in extent, and the limbs and their extremities are affected in a particular manner, so that a slight touch is not felt, but firm pressure over the muscles and the nerve trunks gives rise to acute pain. Besides tenderness, there are acute booring, burning pains in the nerve trunks. The tenderness of the muscles is very characteristic and is well shown by pressing the skin over the anterior tibial muscles, and compare the excessive pain thus produced with the slight discomfort of pressing the skin against the anterior surface of the tibia.

Trophic changes occur sometimes in cases of long standing, in the skin, nails, and joints similar to those occurring in local neuritis. Seldom are the sphinctors affected, and when they are it is due to the disease spreading to the cauda equina or to the spinal cord.

The cause and duration vary much; but in an average case the disease increases from two to four weeks, then remains stationary for a month or two, and then begins to improve. Spontaneous pains become less, but the tenderness still presists; muscular improvements occur in the reverse order of onset, or in other words the muscles which are the first and most affected are the last to recover. In very acute cases, the respiratory muscles may be quickly involved and cause death either directly or by pneumonia. Death may also be caused by other diseases due to alcohol as cirrhosis of the liver, Bright's disease, chronic cerebral meningitis, or from phthisis, a frequent complication. Mental affection, as delirium tremens, may usher in neuritis. These affections may take on the ordinary form of voices jeering at him, or the visional form, as demons or animals in the room or horrid insects crawling over him. Now in these cases of poisonous effects of alcohol in the brain, there is no doubt but that it acts as a direct poison on the cortical cells, in the same way as absinthe will produce epileptic fits by its action on the cortical cells.

We now come to the consideration of lead. Lead may cause not only the peripheral neuritis, but a slower chronic atrophy of the muscles, seen first in the interossei; it is precisely like spinal atrophy, and probably such, but differing in that it does not progress if its causes cease to act, although it is far more enduring than the wrist-drop, it may be permanent, but it does not increase. Lead may cause some form of sclerosis of the cord, usaully slight in degree; it may cause optic nerve atrophy; and many forms of functional disorder may result from it. It may cause tremor, chronic convulsions, like those of epilepsy; and hysteria with its varied manifestations. Neuralgia, sometimes of great severity, may be due to it, and headache is a frequent effect as well as the symptoms of general nervous weakness. In all such cases, in which there is nothing in the symptoms to suggest the cause, this may escape you, unless you are put upon its track either by the occurence of other associated cases of lead poinoning, by the occupation of the patient, or by the presence of its great sign, *the lead line*. This line is said to be blue, but such is not always the case, I have had recently under my observation, a case where the line was not to be seen at all. You may ask why this prominent point of diagnosis is absent? It is the edge of a deposit of sulphide of lead beneath the inner surface of the gum, where this is separated from the teeth even in a very slight degree. The sulphur comes from albuminous substances which decompose them. Sometimes we see a similar deposit on the mucus membrane of the lower lip, when there is tartar on the teeth with which it comes in contact. Tartar contains organic substances mixed with earthy salts from the saliva, and it yields enough sulphur to act on the lead. It is often the case when we find two or three isolated spots, or on the tips of the projections of gums between the teeth, in this line of patients. A year ago a patient was sent me, for muscular wasting. On examination there was atrophy of the muscles of the forearm, but it was the muscles that suffer in lead poisoning, with characteristic wrist-drop as its result. No others were affected, they had lost ferradic irratibility and presented very little voltaic excitability. The excitability to voltaism quickly improves in this state after a short treatment.

Lead when taken into the system in repeated small doses affects the nervous system in the form of loss of power and of sensation in the limbs, accompanied by pains or less frequently there are general cerebral disturbances, with convulsions. Where there is no hereditary tendency yet some seem to have a special proclivity to lead as with many other drugs. No age is exempt, women are more liable than men.

The disease that predisposes to lead poisoning is *Gout*.

It is not a difficult matter to detect lead among plumbers, painters, and other trades who use lead, but in people who are not in contact with lead, it is difficult to detect. In the former case it is usually taken into the mouth by men neglecting to wash their hands before meals, and in the latter through drinking water kept in lead pipes and cisterns, from snuff which has been wrapped in lead paper, from cider or acid stewed fruits kept in porcelain vessels glazed with lead, or from using lotions and washes containing lead. Lead may be absorbed by the alimentary canal or skin, and in animals which have been fed on lead, it has been found after death in the spinal cord.

*Symptoms.*—The chief symptoms which affect the nervous system are paralysis of certain muscles and in other cases cerebral disturbances with mental changes.

Paralysis occurs in two forms, acute and chronic. The acute is the most common, and it represents the typical wrist-drop of lead paralysis, and effects the two sides symmetrically. The first symptom is weakness in extending one finger of one hand, usually the right, and this progresses to other fingers, the thumb, the extensor ossis metacarpi the last affected. There may be no pain or sharp pains along the nerve trunks, with some tenderness of the muscles; while sometimes we see painful spasms or cramps on the muscles. The difficulty is in extending the meta-corpa phalangeal joints. Later the extensors of the wrists become affected, and then the wrist is dropped, and cannot be moved into line with the forearm. In making the examination of the wrist, we should fix the forearm by laying the limb on a table so that the hand hangs over the edge, and then notice if the hand can be raised at all out of its hanging position. All the muscles on the back of the forearm are affected, except the supinator longus, which is rather remarkable for it is supplied from the same nerve, the musculo-spiral, and can be readily made to contract by the patient fixing his elbow against resistance. The flexors of the fingers are not affected, and this apparent weakness is due to their acting at a disadvantage, their muscles being shortened owing to the wrist being fixed by the paralysed extension of the carpus. Later on the muscles of the upper arm may be involved and especially the biceps, branchialis anticus and deltoia. General tremors with hyper-excitability to precussion are sometimes observed with twitching of the muscles. The muscles rapidly waste and show changes to electrical testing; to the faradic current, excitability is diminished or completely lost, while to the galvanic current excitability is increased. and the contraction to the positive pole is obtained with a weaker current than with the negative.

*Pathology.*—The question that now naturally arises is:

"What is the seat of the lesion?" This differs according to whether the symptoms are acute or chronic. In the acute form of double wrist-drop, the changes are essentially those of a peripheral neuritis, having a special selection for the nerves supplying the extensors of the fingers and wrist.

The spinal cord is not as a rule affected, except in the slow chronic form of progressive muscular atrophy. No definite changes have been found in the brain.

Before concluding I wish to make a few remarks on *arsenic.*

Poisoning by small doses of arsenic in occasionally produced either by taking the drug medicinally, or more frequently from cosmetics or sleeping in rooms with arsenical wall papers.

Like lead it causes neuritis, but arsenical neuritis usually affects the legs before the arms, although the latter suffer in severe cases. Moreover its effects on the nerves vary much; the common palsy may be absent and the sensory nerves may suffer much or most and hence the symptoms may be equivocal and even misleading. As a rule the symptoms are acute, and consist of numbness and tingling, and later of sharp burning pains in the limbs, with tenderness along the nerve trunks and hyperaesthesia of the muscles, followed by anaesthesia of the skin and loss of power in the extensors of the wrists and also the toes.

Just as the lines on the gums are the indication of lead, changes on the skin, especially certain forms of pigmentation, constitutes the outward and visible sign of the influence of arsenic. The brown pigmentation begins as small spots, which commence in spots of conjestive redness, and the brown tints succeed the red. It is not so dark as the pigmentation of Addison's disease, and has not the same distribution.

A most important question now arises: How is a differential diagnosis to be made of paralysis due to arsenic, from lead, and alcohol? The absence of a blue line and of any history of poisoning by these two, and the symptoms of ataxia associated with tenderness of the nerve trunks, should help the diagnosis. The paralysis due to arsenic has less tendency to recover than that of lead or alcohol.

